# Hindlimb unloading induces time-dependent disruption of testicular histology in mice

**DOI:** 10.1038/s41598-022-22385-9

**Published:** 2022-10-18

**Authors:** Asima Karim, Rizwan Qaisar, Muhammad Azeem, Josemin Jose, Gopika Ramachandran, Zeinab Mohamed Ibrahim, Adel Elmoselhi, Firdos Ahmad, Wael M. Abdel-Rahman, Anu V. Ranade

**Affiliations:** 1 Department of Basic Medical Sciences, College of Medicine, University of Sharjah, 27272 Sharjah, United Arab Emirates; 2 Department of Applied Physics and Astronomy, College of Sciences, University of Sharjah, 27272 Sharjah, United Arab Emirates; 3 Sharjah Institute for Medical Research, University of Sharjah, 27272 Sharjah, United Arab Emirates; 4 Department of Medical Laboratory Sciences, College of Health Sciences, University of Sharjah, Sharjah, United Arab Emirates

**Keywords:** Cell biology, Anatomy, Endocrinology

## Abstract

Mechanical unloading of the body in the hindlimb unloaded (HU) mice induces pathology in multiple organs, but the effects on testes are poorly characterized. We investigated the histology and Raman spectroscopy of the mouse testes following HU condition. We divided male, c57BL/6j mice into ground-based controls or experimental groups for two and four weeks of HU. The testes tissues were dissected after euthanasia to investigate histological and Raman spectroscopic analysis. We found an HU-induced atrophy of testes irrespective of the time duration (p < 0.05). Histological analysis revealed that the HU induced epithelial thinning, luminal widening, and spermatozoa decline in the seminiferous tubules of the mouse testes. In addition, we found a thickening of the epididymal epithelia and tunica albuginea. These changes were accompanied by a generalized depression in the Raman spectra, indicating an altered concentration and/or orientation of several molecules. We also report reduced signal intensities of hydroxyproline and tryptophan, potentially contributing to testicular pathology during HU. Taken together, our findings indicate that the two or four weeks of HU induce disruption of testicular architecture and molecular phenotypes. Our results may have implications for understanding and/or treating male infertility associated with prolonged bed rest and spaceflight.

## Introduction

The human body is evolutionarily designed to live in earth’s gravity. However, conditions of reduced gravity, such as spaceflight and prolonged bed rest, negatively affect various physiological functions of the body^1^. Male reproductive organs are specifically vulnerable to damage due to reduced gravity^2^. This is partly due to the cephalic redistribution of body fluids and the decline of other body systems, including cardiovascular and musculoskeletal systems. Reduced gravity induces several structural and functional alterations in testicular tissues. For example, reduced spermatogenesis is a common finding in reduced gravity conditions, as the early stages of spermatogenesis are susceptible to compromised testicular blood supply^3,4^. Similarly, several histopathological alterations are observed in testicular tissues under reduced gravity conditions.

The ground-based mouse model of hindlimb unloading (HU mice) mimics several physiological and pathological alterations in body systems during reduced gravity^5–7^. These include skeletal muscle wasting, osteoporosis, cardiovascular deconditioning, orthostatic hypotension, intestinal dysbiosis, elevated oxidative stress, and compromised immunity^5–7^. The HU-induced detriment of several body features has been thoroughly characterized over the past four decades^8,9^. Among different body systems, the male reproductive system may be of primary relevance in dictating the generalized health and male phenotype. Specifically, testes secrete androgens, which have systemic effects on multiple organs due to their anabolic actions. Consequently, a thorough characterization of testicular morphology in the HU may help understand the detrimental effects of bed rest and simulated microgravity on male reproductive system. However, only a handful of studies have investigated the male reproductive system in HU conditions. For example, seven days of HU is shown to lower testosterone levels and impair spermatogenic function in mice^10^. Further, a significant reduction in the expression of androgen receptors is also reported following seven days of HU, which is associated with irreversible pathological damage as the duration of HU further increases^11,12^. This decline in the male reproductive hormones may cause systemic effects on the health of several body systems, warranting the necessity of restoring testicular function^13,14^. However, despite the adequate evidence of male reproductive suppression during unloading, the histopathological changes in testes are poorly characterized. Specifically, the timing of the induction and exacerbation of testicular pathology during HU is not known.

This study primarily aims to bridge this gap in literature by investigating the histopathological changes in the mouse testes under HU conditions. We took a time-dependent approach to measure the alterations in testicular histology with increasing duration of HU. Based on a carful literature search and our pilot investigations, we chose the durations of two and four weeks of HU. These durations have translational potential since bedrest of comparable durations in humans result in dysfunction of several body systems, including reproductive system^15,16^. In addition to characterizing testicular morphometry, we also evaluated the global molecular phenotypes of HU mice testes using Raman Spectroscopy. We hypothesized that the testicular tissues would show a time-dependent detriment in histology and molecular phenotype during HU conditions.

## Materials and methods

### Animals and HU conditions

We randomly assigned 4-month-old, male c57BL/6j mice into ground-based controls and HU mice (n = 8–12/group). The HU mice were further divided into two subgroups based on two- and four-weeks duration of HU. The mice were kept under controlled environmental conditions (20 ± 1 °C, with light/dark periods of 12 h each) with food (standard chow diet for mice) and water provided *ad-libitum*. The HU mice were suspended in specially designed cages, as described previously^17,18^. At the end of the experiments, mice were euthanized via cervical dislocation, and the testes were immediately harvested and weighed. Tissue was either fixed in Bouin’s and processed for histopathological staining (Hematoxylin & Eosin and Masson’s trichrome) or snap-frozen for Raman Spectroscopy analysis. The experimental protocol was approved by the Animal Care and Use Committee of the University of Sharjah in agreement with accepted international standards. All experiments were approved and conducted in accordance with the relevant guidelines and approvals set by the University of Sharjah and followed the recommendations in the ARRIVE guidelines.

### Testicular and epidydimal morphometry

Following the dissection of gastrocnemius muscle and reproductive organs, testis and the epididymis were fixed in Bouin’s solution and stored in 70% ethanol and subjected to paraffin embedding. For light microscopic examination, the ethanol-fixed tissue was sectioned at 4 µm and further stained with hematoxylin and eosin (H&E) and Masson’s trichrome^19^. Morphometric analysis was done as described previously by us^20^. All stained sections were individually examined at different magnification using digital imaging camera attached microscope (Olympus BX 60). The images were captured and imported on ImageJ software for analysis. Standard luminal diameter was measured by averaging two different distances (180° vertical and 180° horizontal) from cross-sections of seminiferous tubules from 10 randomly selected seminiferous tubules per animal regardless the stage of the cycle. The standard epithelial height was calculated as the average of five different measurements from the basal membrane to the tubular lumen in cross sections of 10 randomly selected seminiferous tubules. Both the epithelial height and the luminal diameter was obtained from the same seminiferous tubules. Likewise, the epididymal epithelial height was determined from the caput region by measuring five different lengths of a tubule from its basal lamina to apical end of the cells excluding its stereocilia. The density of the spermatozoa was measured using image J software (available at https://imagej.nih.gov/ij/download.html). The mean area percentage of collagen fibers content was analyzed using the Image-Pro Plus program, while the spermatogenetic activity evaluation was done using Johnson’s score^21^.

### Raman spectroscopy

The experimental Raman spectra were obtained by using Renishaw inVia confocal Raman microscope. Following the euthanasia, the testes tissues were snap-frozen for analysis. We collected ten spectra from randomly selected locations of each sample to obtain the average. Each recording involved exposure of a 50 µm tissue segment for 10 s to a 785 nm laser of 1% intensity (14 mW), as described previously^18^. The spectral range was kept between 100 and 1200 cm^−1^, representing the peaks for biological molecules. All spectra were collected at 500 cm^−1^ intervals and then stitched together to obtain the full spectrum^18^.

### Statistical analysis

All numerical values are presented as mean ± SEM, and the comparisons among the groups were performed by one-way analysis of variance (ANOVA) and Turkey’s multiple comparison tests, with a single pooled variance. Data were analyzed using GraphPad Prism 9 (GraphPad Software, La Jolla, CA), and p < 0.05 was considered statistically significant.

## Results

### Mice characterization

**Figure 1:**
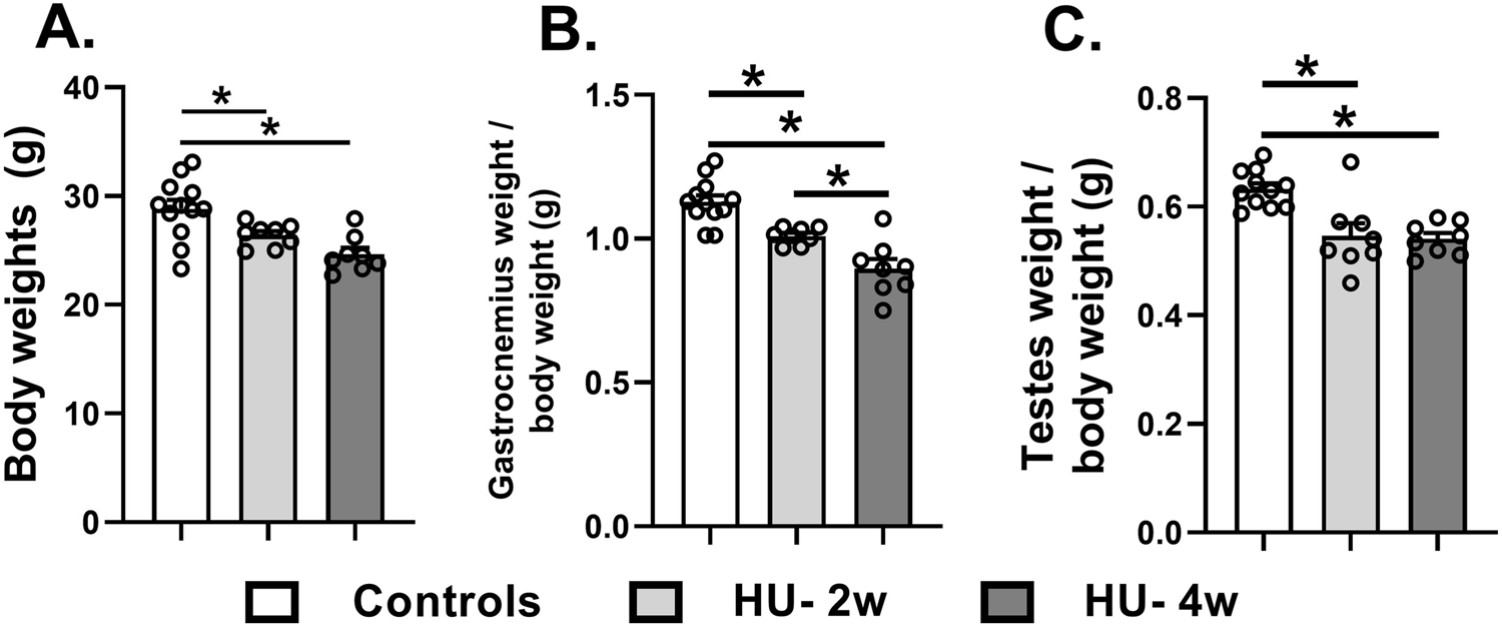
Bodyweight (**A**), and the weights of gastrocnemius muscle (**B**), and testes normalized to body weights (**C**) in ground-based controls and HU mice suspended for 2 and 4 weeks. Data is represented as mean ± SEM (n = 8–12/group). One-way analysis of variance, *p < 0.05.

The HU resulted in reduced body weights of the mice suspended for two (mean ± SD = 26.3 ± 0.9 g and four (mean ± SD = 24.9 ± 2.1 g) weeks when compared to ground-based controls (mean ± SD = 29.1 ± 2.7 g) (all p < 0.05) (Fig. 1A). We first validated the mouse model by showing time-dependent atrophy of gastrocnemius muscle normalized to body weights in HU mice suspended for two (mean ± SD = 1.01 ± 0.03) and four (mean ± SD = 0.89 ± 0.088) weeks, when compared to ground-based control mice (mean ± SD = 1.13 ± 0.072 g) (all p < 0.05) (Fig. 1B). Next, we found that the normalized testicular weight was significantly lower in both groups of HU mice (mean ± SD = 0.549 ± 0.084 and 0.541 ± 0.026 for two and four weeks of HU, respectively) than ground-based controls (mean ± SD = 0.632 ± 0.037) (all p < 0.05) (Fig. 1C).

### Testicular morphometry

**Figure 2:**
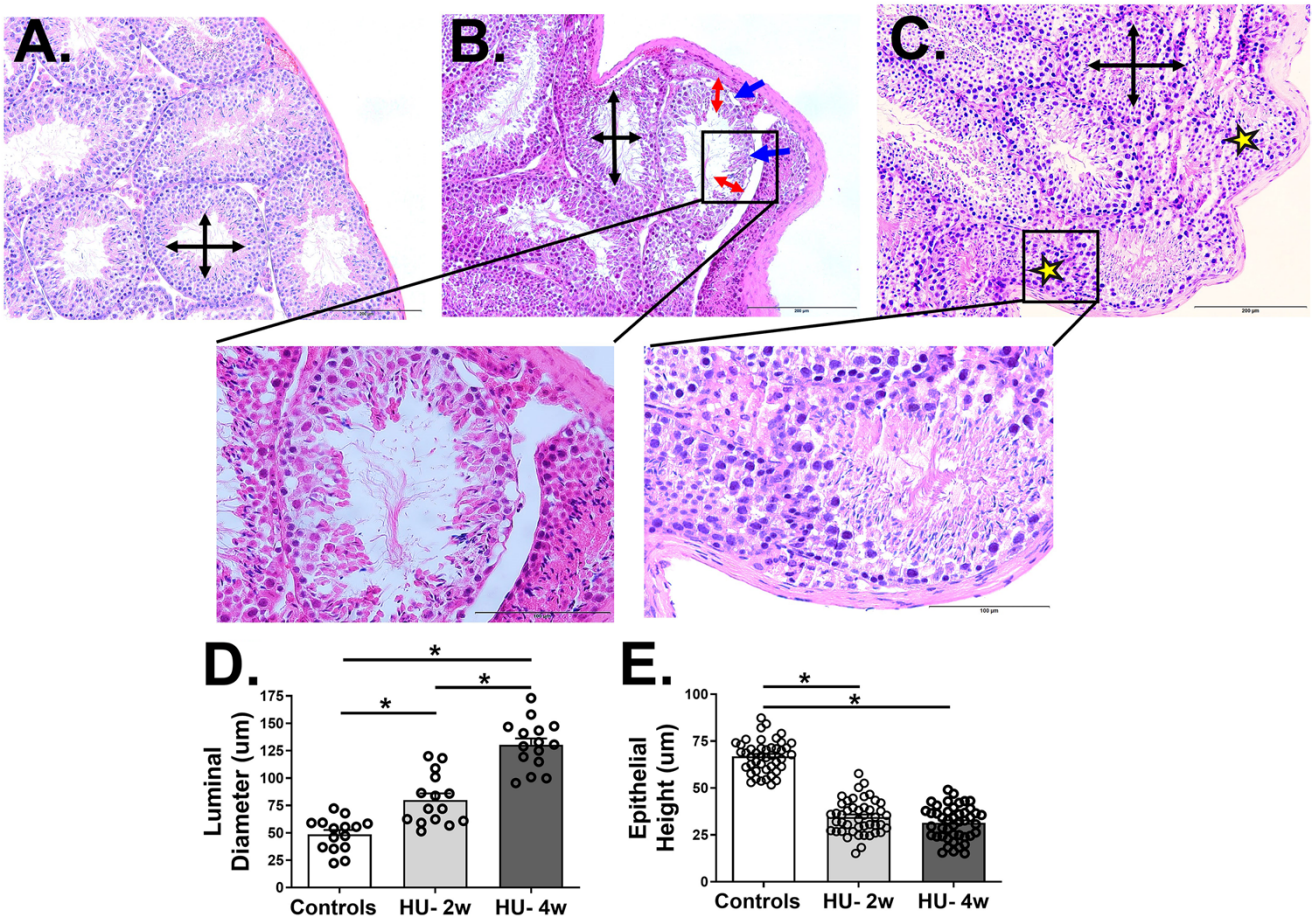
Histopathological features of testes tissues from ground-based controls (**A**) and HU mice suspended for two (**B**) and four (**C**) weeks. The image analysis shows the tubular diameter (**D**) and epithelial height (**E**) in the three groups of mice. The luminal diameter (black arrows) reduced epithelial height (red arrows), vacuolation (blue arrows), and the areas of necrosis (stars) are marked in the images. Data is represented as mean ± SEM (n = 15 images/group for 2D and 45 images/group for 2E, 8–12 mice/group). Images (**A**,**B**) were taken at 20 × magnification, while the zoomed-in inset images were taken at 40 × magnification. One-way analysis of variance, *p < 0.05.

We next investigated the microscopic analysis of the testes in the three groups of mice. A significant deterioration of the testicular histology was observed in the HU mice in a time-dependent manner. The ground-based control mice exhibited a homogenous testicular architecture with more than 90% of the seminiferous tubules showing normal morphology, well-developed spermatogenic cells, and dense spermatozoa (Fig. 2A). However, two weeks of HU resulted in a disrupted tubular architecture, characterized by epithelial thinning and the appearance of vacuolation (Fig. 2B). The HU for four weeks also resulted in necrotic tissues in the seminiferous tubules (Fig. 2C). Image analysis revealed increased standard luminal diameter in mice during HU conditions for two (mean ± SD = 80.04 ± 22.87 µm) and four (mean ± SD = 130.6 ± 22.2 µm) weeks than ground-based controls (mean ± SD = 48.69 ± 14.92 µm) (all p < 0.05). Similarly, we found a reduction in epithelial height in both groups of HU mice (mean ± SD = 34.57 ± 8.99 µm and 31.47 ± 8.85 µm for two and four weeks of HU, respectively) than controls (mean ± SD = 67.12 ± 9.22 µm) (all p < 0.05) (Fig. 2D,E).

**Figure 3:**
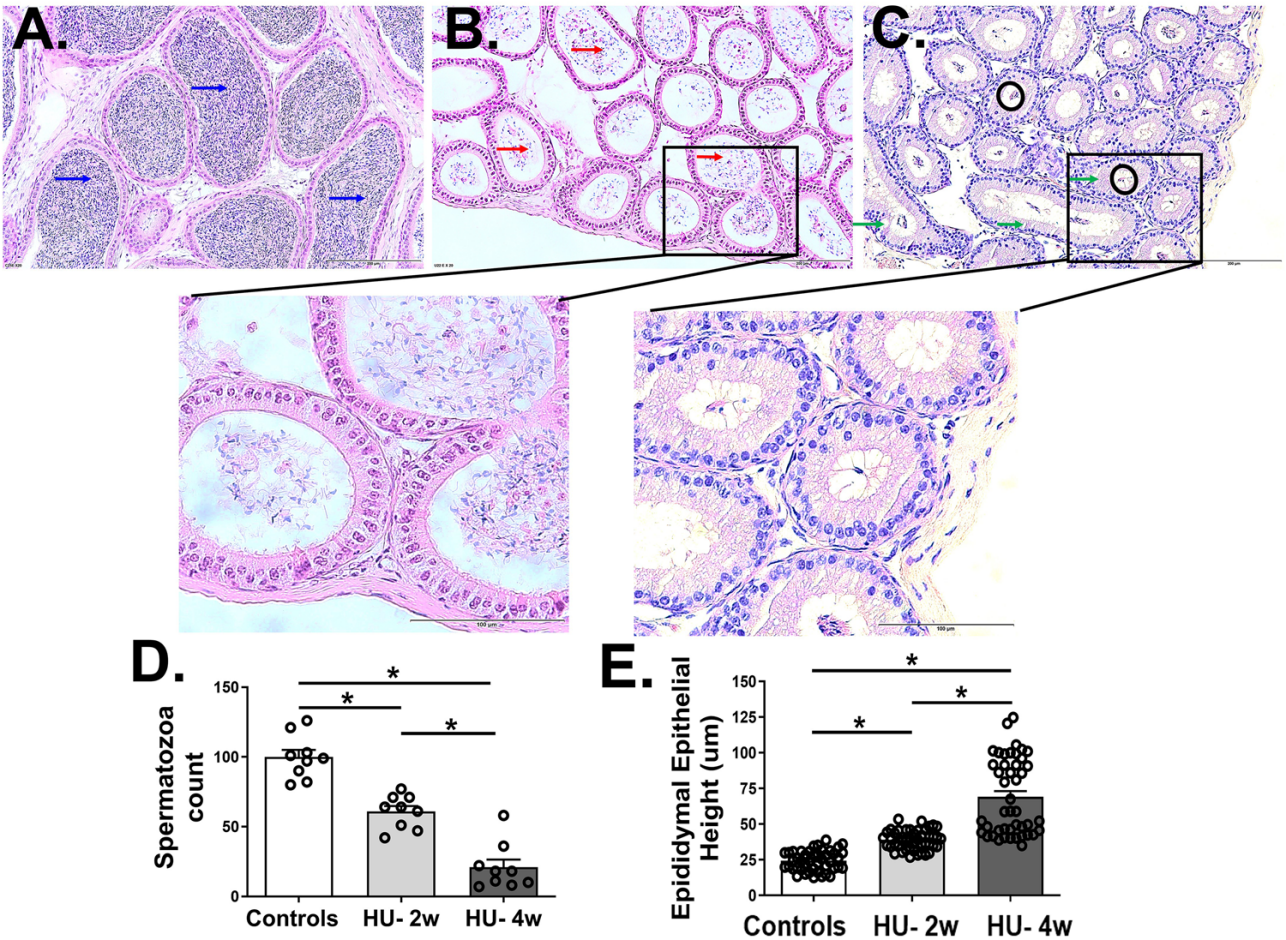
Histopathological features of epididymal tissues from ground-based controls (**A**) and HU mice suspended for two (**B**) and four (**C**) weeks. The image analysis shows the spermatozoa count (**D**) and epididymal epithelial height (**E**) in the three groups of mice. The high spermatozoa density (blue arrows), spermatozoa thinning and tubular debris (red arrows), retraction of the spermatozoa into the central portions of the tubular lumen (black circle), and the areas of vacuolation (green arrows) are marked in the images. Data is represented as mean ± SEM (n = 9 images/group for (**D**) and 45 images/group for (**E**), 8–12 mice/group). Images (**A**–**C**) were taken at 20 × magnification, while zoomed-in inset images were taken at 40 × magnification. One-way analysis of variance, *p < 0.05.

We next investigated the epididymal tissues of the three groups of mice. The spermatozoa density was significantly reduced in both groups of HU mice (mean ± SD = 60.89 ± 11.89 and 20.89 ± 16.58 for two and four weeks of HU, respectively) when compared to control mice (mean ± SD = 99.89 ± 15.66) (p < 0.05) (Fig. 3A–D). In addition, we also observed the retraction of the spermatozoa into the central portions of the tubular lumen. We also found an increase in the epididymal epithelial height with two (mean ± SD = 38.94 ± 6.81 µm) and four (mean ± SD = 68.86 ± 26.92 µm) weeks of HU when compared to ground-based control mice (mean ± SD = 24.25 ± 6.99 µm) (all p < 0.05) (Fig. 3E).

**Figure 4:**
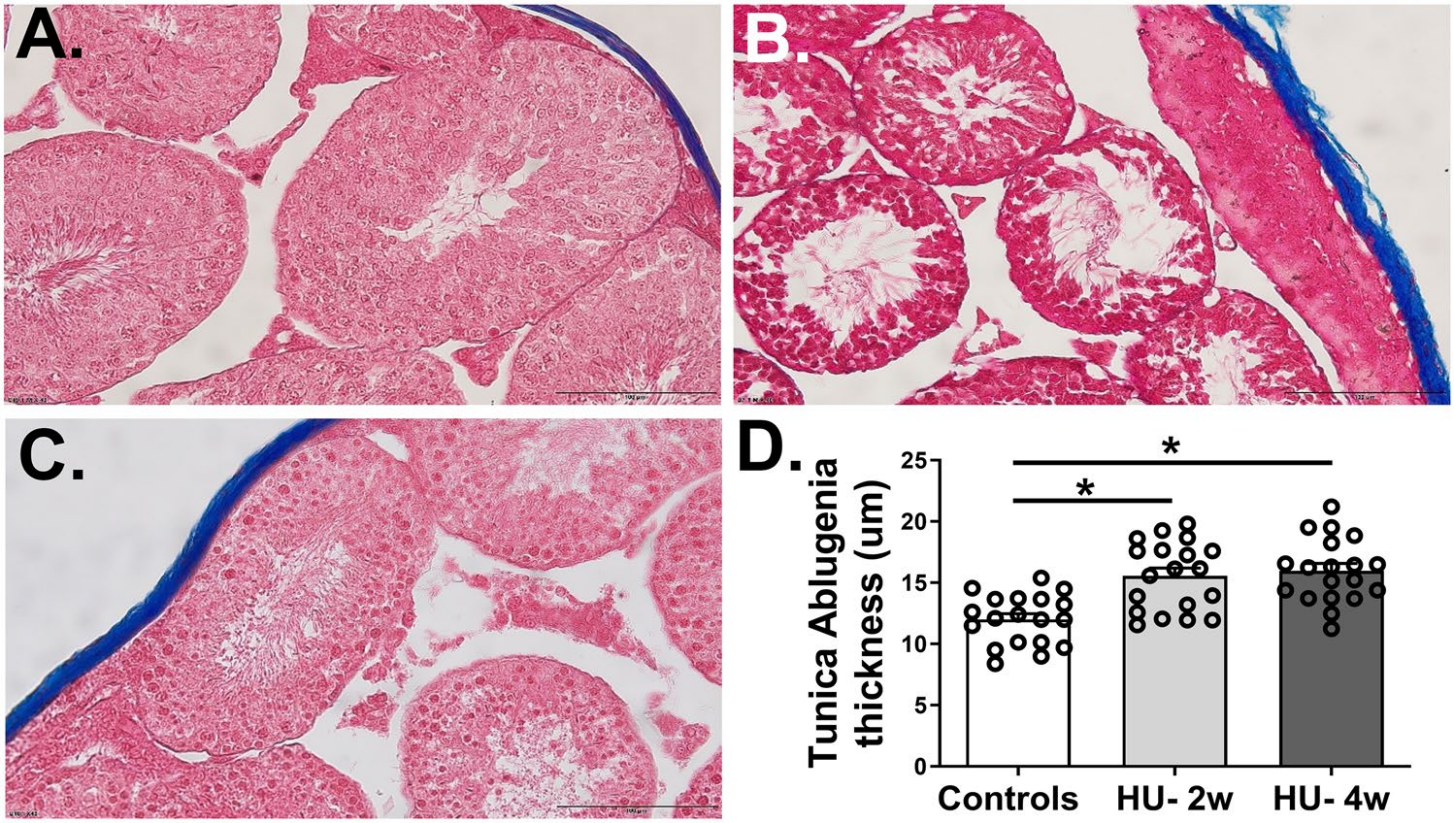
Histopathological features of tunica albuginea of testes stained with Masson’s trichrome from ground-based controls (**A**) and HU mice suspended for two (**B**) and four (**C**) weeks. The image analysis shows the thickness of the tunica albuginea (**D**). Data is represented as Mean ± SEM (n = 19 images and 8–12 mice/group). All images were taken at 40 × magnification, One-way analysis of variance, *p < 0.05.

We next used Masson’s trichrome to measure the thickness of tunica albuginea covering the anterior aspect of the testis in three groups of mice. Three samples from each group with four sections per group and four fields from each section were selected. The images were examined using Olympus BX 60 microscope, captured with digital imaging camera, and quantified using ImageJ software to analyze the thickness. Compared to control mice (mean ± SD = 12.01 ± 2.04 µm), the HU resulted in a significant thickening of tunica albuginea at two (mean ± SD = 15.55 ± 2.88 µm) and four (mean ± SD = 15.97 ± 2.61 µm) weeks timepoints (both p < 0.05) (Fig. 4).

### Raman spectroscopy analysis

**Figure 5:**
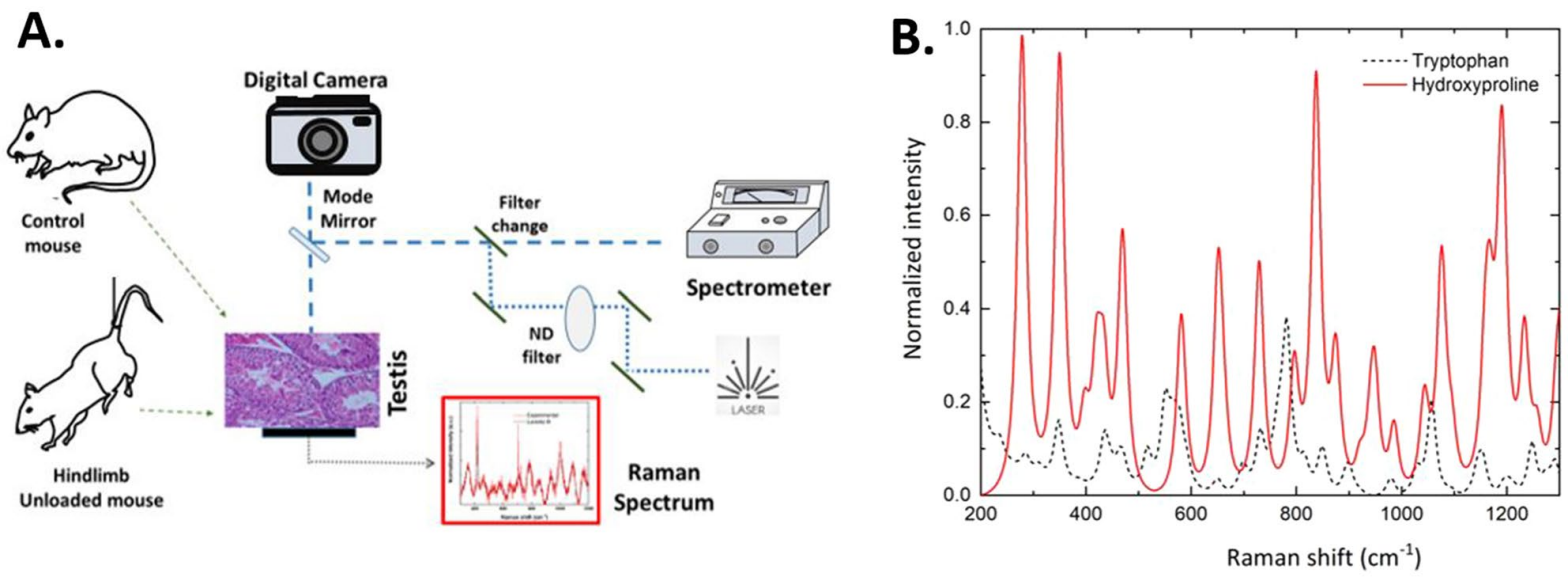
Schematic representation of the experimental procedure for collecting Raman spectroscopic data (**A**) and the calculated Raman spectra for tryptophan and hydroxyproline molecules and their average spectra (**B**) from the experimental mice.

Raman spectra were obtained from snap-frozen testicular tissues of the three groups of mice (Fig. 5A). We first calculated the spectra for the molecules of tryptophan [C_11_H_12_N_2_O_2_] and hydroxyproline [C_5_H_9_NO_3_], which are among the most abundant amino acids in the biological tissues^22^. The molecular structures were obtained from the PubChem database^23^. The theoretically calculated spectra for both molecules (Fig. 5B). Our model showed that the band at 351 cm^−1^ was a cumulative effect of the rocking vibrational modes of H–N–H, H–C–H, and C–O–H bonds of tryptophan and O–H bonds of hydroxyproline. The band at 459 cm^−1^ in the experimental agreed with the model, which showed a peak at a slightly higher frequency, 468 cm^−1^. It emerged due to C–C ring vibrations of hydroxyproline. The stretching vibrations of N–H and H–N–H bonds of tryptophan gave rise to a Raman band at 526 cm^−1^ in the experimental spectrum. However, it appeared to be more robust when compared to the corresponding calculated bands at 516 cm^−1^ and 552 cm^−1^.

**Figure 6:**
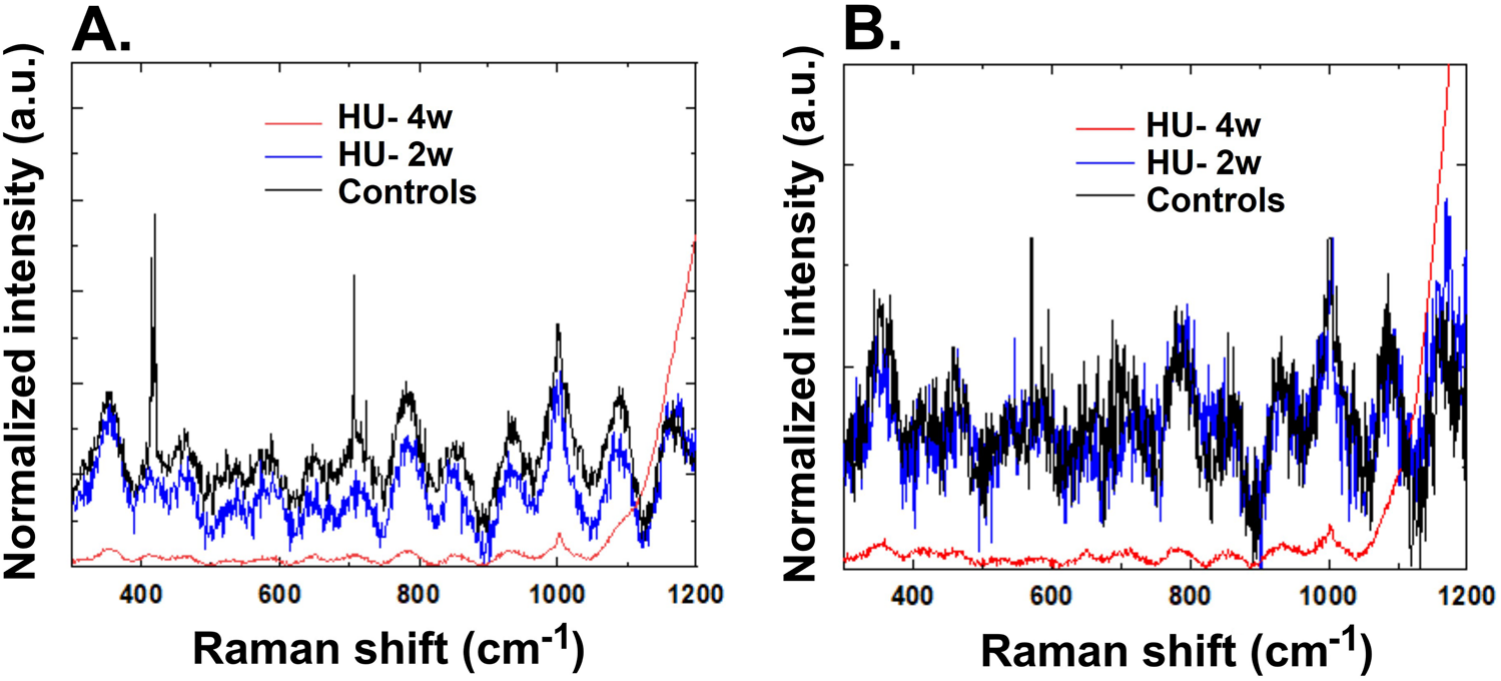
Raman spectra from the seminiferous tubules (**A**) and tunica albuginea (**B**) of ground-based controls and HU mice suspended for two and four weeks (n = 8–12/group).

We next investigated the global Raman spectra of seminiferous tubules and tunica albuginea from the three groups of mice (Fig. 6A,B). We found that two weeks of HU resulted in generalized depression of the signal intensity across the whole frequency range. The four weeks of HU further reduced the signal intensity from the mice tissues. These findings indicate deterioration of the global molecular phenotypes of the mouse seminiferous tubules and tunica albuginea due to HU.

## Discussion

To our knowledge, this is the first study investigating the time-dependent alterations in histopathology and Raman spectra in testicular tissues of HU mice. We investigated the pathological effects of HU on mouse testes in a time-dependent manner. Our findings indicate that the HU results in a significant disruption of testicular architecture, including the expansion of luminal diameter, epithelial thinning, and reduced spermatozoa density. A depression of global molecular phenotype accompanies these changes. We also report a time-dependent effect as the four weeks of HU induced a more severe testicular disruption than two weeks of HU.

The data about testicular histopathology in HU rodents are scarce. A previous study elaborated on loosening on seminiferous tubules along with presence of necrosis^11^. Additionally, they reported interstitial edema and the disappearance of germ cells in the testes. However, this study was conducted on rats. Due to limitations in creating transgenic rat models, the relative scarcity of mechanistic data from rats may reduce the translational potential of such findings in humans. Consequently, our data from mice may be more relevant to clinical studies than data from rats. Our findings of epithelial thinning, reduced spermatozoa density, and areas of necrosis are consistent with previous findings. While we did not find relevant data from clinical studies due to constraints of obtaining testes biopsy, several lines of evidence are consistent with our findings. For example, spaceflight and prolonged bed rest in humans result in reduced circulating testosterone, indicating potential damage to testicular tissues^24^. Similarly, sedentary lifestyles and physical inactivity are associated with infertility in men^25^. This indirect data indicates the potential involvement of testicular disruption in humans during unloading conditions. Thus, the HU mice at least partly mimic the features of reproductive suppression reported in physically inactive humans.

Our findings recapitulate the age-related degeneration of mouse testes. Specifically, the aged mice show a widening of the seminiferous tubular diameter, epithelial thinning, and reduced spermatozoa density^26^. Thus, the HU may induce an accelerated aging phenotype in the mouse testes. This consequence of physical inactivity is evident in multiple body organs, which mimic age-related degeneration during HU^1,5^.

We also observed a time-dependent disruption of mouse testes during HU. This sequential detriment of testicular histology indicates that the suppression of the male reproductive system is a continuous process during inactivity. These findings may help optimize timely interventions of infertility during bed rest in humans.

Raman spectra indicated a robust decline in the concentration of tryptophan and hydroxyproline in a time-dependent manner. Tryptophan is implicated in testosterone production and spermatogenesis in testes^27^. Thus, a reduced expression of tryptophan is consistent with histopathological disruption, including the reduced density of spermatozoa in the HU testes. Hydroxyproline is an essential component of collagen proteins in biological tissues. Among its multiple functions, it is involved in stabilizing collagens^28^. Thus, the reduced hydroxyproline in HU testes may indicate instability of collagens, which may contribute to testicular pathology. The analysis of HU testes also revealed a generalized depression in the spectral peaks of all intensities. This finding indicates a reduced concentration and/or altered orientation of global molecular phenotype, which agrees with the histopathological findings. Additionally, the spectral peaks above 1000 cm^−1^, which indicate the C–H vibration, were suppressed in the HU testes, showing the breakdown of several amino acid molecules. The reduction was time-dependent with a higher suppression of Raman spectra at four weeks, when compared to two weeks of HU in mice testes. We have previously shown that similar alterations in Raman spectra correspond to pathology of muscle skeletal muscle during HU conditions^6,18^. Specifically, we observed conformational changes and/or reduced content of several amino acids accompanying the suppression of Raman spectra in the skeletal muscle of HU mice^6,18^. The amino acids have a potential protective effect against testicular injuries^29^. Thus, our observation of suppressed Raman spectra and testicular histopathology is consistent with literature and indicate the potential efficacy of Raman spectra in diagnosing testicular pathology.

Taken together, our findings indicate that two and four weeks of HU result in the degeneration of mouse testes. Several characteristics alterations were observed in the histological architecture of testicular tissues, involving seminiferous tubules, epithelial thickness, and spermatozoa density. These changes were accompanied by global suppression of Raman spectra, indicating an altered concentration and/or orientation of several molecules. Further studies are required to investigate the mechanistic link between HU and testicular pathology.

## Data Availability

The data is available from the corresponding author upon reasonable request.
